# The Association of MMP-2, MMP-9, and MMP-13 Gene Polymorphisms With Knee Osteoarthritis in the Greek Population

**DOI:** 10.7759/cureus.65379

**Published:** 2024-07-25

**Authors:** Christos Milaras, Panagiotis Lepetsos, Andreas Pampanos, Eustathios Kenanidis, George A Macheras, Despoina Mavrogianni, Dimitra Dafou, Michael Potoupnis, Eleftherios Tsiridis

**Affiliations:** 1 4th Orthopedic Department, KAT Hospital, Athens, GRC; 2 Medical Genetics Department, "Alexandra" General Hospital, Athens, GRC; 3 Academic Orthopedic Department, Papageorgiou General Hospital, Aristotle University of Thessaloniki, School of Medicine, Thessaloniki, GRC; 4 Center of Orthopedic and Regenerative Medicine - Center of Interdisciplinary Research and Innovation, Aristotle University of Thessaloniki, School of Medicine, Thessaloniki, GRC; 5 1st Obstetrics and Gynecology Department, "Alexandra" General Hospital, National and Kapodistrian University of Athens, Medical School, Athens, GRC; 6 Biology Department, Aristotle University of Thessaloniki, Thessaloniki, GRC

**Keywords:** knee, mmp-2, mmp-9, mmp-13, single nucleotide polymorphism, metalloproteinases, osteoarthritis

## Abstract

Introduction

Primary knee osteoarthritis (OA) is a multifactorial degenerative joint disorder characterized by articular cartilage degradation. Matrix metalloproteinases (MMPs) have been reported to play a vital role in OA pathogenesis, significantly contributing to extracellular matrix (ECM) catabolism. The purpose of this study is to investigate the association of MMP-2 -1575G/A (rs243866), MMP-9 836A/G (rs17576), and MMP-13 -77A/G (rs2252070) gene polymorphisms with knee OA in the Greek population.

Methods

One hundred patients (24% males, mean age: 68.3 years) with primary knee OA were included in the study along with 100 controls (47% males, mean age: 65.2 years). Genotypes were identified through polymerase chain reaction (PCR) and restriction fragment length polymorphism (RFLP) technique. Allelic and genotypic frequencies were compared between patients and controls.

Results

The MMP-13 -77A/G polymorphism was significantly associated with knee OA in the crude analysis (P = 0.008). After binary logistic regression analysis, the dominant model of the MMP-13-77A/G (AG + GG versus AA) was found to be associated with increased risk for knee OA (odds ratio (OR) = 2.290, 95% confidence interval (95%CI) = 1.059-4.949, P= 0.035). Compared to the A allele, the G allele in the MMP-13rs2252070 locus was a predictive factor for knee OA (OR = 2.351, 95%CI = 1.134-4.874, P= 0.022). No significant associations were detected for the MMP-2 -1575G/A and MMP-9 836A/G polymorphisms (P > 0.05).

Conclusions

The present study shows that the MMP-2 -1575G/A and MMP-9 836A/G polymorphisms are not significantly associated with primary knee OA in the Greek population. The MMP-13 -77A/G was found to be a significant risk factor for knee OA in the Greek population. Additional research is needed to verify this association in larger and different populations, in different joints, to elucidate the role of this single nucleotide polymorphism (SNP) in OA pathogenesis.

## Introduction

Primary knee osteoarthritis (OA) is a degenerative joint disorder that affects millions of patients worldwide. Knee OA is a complex disease characterized by articular cartilage degradation, synovial inflammation, osteophyte formation, and subchondral bone sclerosis. The cumulative effect of these processes ultimately results in the characteristic cartilage erosion, pain, and functional impairment seen in primary knee OA. Its pathogenesis involves a complex interplay of genetic, mechanical, and biochemical factors [1].

Matrix metalloproteinases (MMPs) are a family of zinc-dependent endopeptidases that play pivotal roles in tissue remodeling, embryogenesis, angiogenesis, cellular proliferation and apoptosis, cytokine activity, wound healing, and various pathological processes [2]. These enzymes are involved in the degradation of extracellular matrix (ECM) components, thus influencing the structure and function of tissues throughout the body. MMP activity is tightly regulated at multiple levels to prevent excessive tissue degradation. The transcription of MMP genes is influenced by cytokines, growth factors, and tissue-specific signals [3]. Dysregulation of MMP activity can result in pathological tissue remodeling, contributing to degenerative tissue changes in OA [4].

Chondrocyte-derived MMPs play a dual role in the knee joint, contributing to normal joint homeostasis and the pathogenesis of knee OA. In normal knees, they are produced at low levels; however, this production is enhanced in osteoarthritic tissues [5]. Elevated MMP activity, particularly MMP-1 and MMP-13, contributes to the degradation of articular cartilage [6]. Increased MMP activity can produce pro-inflammatory cytokines, contributing to synovitis and the release of further degradative enzymes. MMPs produced by activated osteoblasts located within subchondral cysts influence the remodeling of subchondral bone, contributing to the formation of osteophytes seen in OA [7]. MMPs can affect bone turnover, leading to subchondral sclerosis and alterations in bone morphology [8].

MMP production is regulated at the genetic level, as most MMP genes are only expressed during tissue remodeling. The regulation of MMP gene expression involves a complex interplay of transcription factors, signaling pathways, and epigenetic modifications. A single nucleotide polymorphism (SNP) is a common genetic variation that contributes to genetic diversity at a single nucleotide. The identified SNPs in genes encoding MMPs influence MMP expression, production, and enzymatic activity [9].

The MMP-2 -1575G/A polymorphism (rs243866) results from the substitution of guanine for adenine at position 1575 of the gene promoter and is located very close to a potential estrogen receptor binding site. The G allele acts as a transcriptional enhancer, while the A allele reduces the MMP-2 transcriptional activity [10]. This polymorphism has been associated with hypertension, heart failure, myocardial infarction, childhood obesity, colon cancer, prostate cancer, primary ovarian insufficiency, and open-angle glaucoma [11].

The MMP-9 gene is located on chromosome 20q11.2-q13.1 and consists of 13 exons. The MMP-9 c.836A>G (rs17576) polymorphism is located in exon 6 and results from adenine to guanine substitution at position 836, affecting the substrate binding domain of the MMP-9 enzyme, substituting an uncharged amino acid (glutamine) for a positively charged amino acid (arginine) (p.Gln279Arg). This polymorphism probably alters the conformation of the protein, leading to a change in the substrate binding and MMP-9 enzymatic activity [12]. The rs17576 polymorphism has been associated with a plethora of diseases such as hypertension, atherosclerosis, coronary artery disease, diabetes mellitus, asthma, Parkinson's disease, glaucoma, and malignant tumors (mesothelioma, nasopharyngeal carcinoma, lung cancer, and cervical cancer) [13,14].

SNPs in the promoter region of the MMP-13 gene can influence gene expression and, in turn, affect the production, structure, and function of the MMP-13 enzyme. The MMP-13 -77A/G polymorphism (rs2252070) of the promoter region results from the substitution of adenine for guanine at position 77. The presence of the A allele is associated with a double higher transcriptional activity of the MMP-13 gene in comparison to the G allele [15]. The specific SNP has been identified as a risk factor for several types of cancer and chronic inflammatory diseases [15].

Based on the potential role of MMPs in knee OA pathogenesis [4,8], we assumed that the existence of SNPs in MMP genes may affect their production and enzymatic activity, thus contributing to OA progression. The purpose of this study is to investigate the effect of gene polymorphisms MMP-2 -1575G/A (rs243866), MMP-9 836A/G (rs17576), and MMP-13 -77A/G (rs2252070) in the risk of knee OA in the Greek population.

## Materials and methods

The study was approved by the Ethics Review Committee of Papageorgiou General Hospital (197/17.6.2020). Written informed consent was obtained from all participants before the collection of blood samples. A prospective, non-randomized case-control study was conducted in our institution between July 2020 and May 2022. The patient group included adults, with primary knee OA and a score ≥ 2 on the Kellgren and Lawrence (K-L) scale [16]. All participants underwent knee clinical examination and anteroposterior knee X-rays to diagnose knee OA. Patients were excluded in the presence of secondary knee arthritis, including rheumatoid, inflammatory, post-traumatic, or septic arthritis. The control group consisted of adults without any symptoms or clinical signs of knee OA, including knee pain, swelling, tenderness, and limited motion, with a score < 2 on the K-L scale. The demographic and radiological data of the participants and their body mass index (BMI) were also recorded.

Venous blood sample (5 mL) was collected from each participant and stored at -20°C in sodium citrate tubes. DNA was isolated with a DNA extraction kit (QIAamp DNA Blood Midi Kit, QIAGEN Inc., Valencia, CA) according to the manufacturer's instructions. The quality and quantity of DNA were checked using an ultraviolet-visible (UV-vis) spectrophotometer (Nanodrop, Thermo Fisher Scientific, Waltham, MA). Genotyping was carried out by standard polymerase chain reaction (PCR) and restriction fragment length polymorphism (RFLP), as previously described [17-19].

For the -1575G/A SNP (rs243866), the MMP-2 gene was amplified by PCR using the sense primer 5’-GTCTGAAGCCCACTGAGACC-3’ and the antisense primer 5’-CTAGGAAGGGGGCAGATAGG-3’ following an initial denaturation step at 94°C for eight minutes and amplification of 40 cycles in a three-step reaction that consisted of denaturation at 94°C for 60 seconds, annealing at 56°C for 30 seconds, and extension at 72°C for 50 seconds, with a final extension step at 72°C for five minutes. Then, 8 μL of PCR product (175 bp) was digested with 3U NlaIII (New England Biolabs, Inc., Ipswich, MA), 2 μL 10× buffer, and 8 μL ddH2O for 16 hours at 37°C. Transition of G à A at position -1575 of the promoter region creates a NlaIII digestion site, whereas the PCR product amplified from the wild type is resistant to NlaIII digestion. The undigested wild type PCR products and the cleavage products were separated by electrophoresis on a 2% agarose gel containing ethidium bromide (EtBr) and further visualized under ultraviolet light. Digestion of the PCR products yielded bands of 175 bp in GG-homozygotes, 112 and 63 bp in AA-homozygotes, and all three bands (175, 112, and 63 bp fragments) in heterozygotes.

For the 836A/G SNP (rs17576), the MMP-9 gene was amplified by PCR with the sense primer 5’- TCACCCTCCCGCACTCTGG-3’ and the antisense primer 5’- CGGTCGTAGTTGGCGGTGG-3’, using an initial denaturation step at 95°C for five minutes, followed by 30 cycles of amplification in a three-step reaction that consisted of denaturation at 94°C for 30 seconds, annealing at 66°C for 60 seconds, and elongation at 72°C for 60 seconds, with a final extension step at 72°C for 10 minutes. Then, the PCR product (300 bp) was digested with Msp1 (New England Biolabs, Inc.) for 16 hours at 37°C. A single fragment of 300 bp was identified as AA-homozygotes; three fragments of 300, 170, and 130 bp were identified as AG-heterozygous; and two fragments of 170 and 130 bp were identified as GG-homozygotes. All products were separated by electrophoresis on a 3% agarose gel stained with EtBr, and they were visualized under ultraviolet light.

For the -77A/G, the MMP-13 gene was amplified by PCR with the sense primer 5’-GATACGTTCTTACAGAAGGC-3’ and the antisense primer 5'-GACAAATCATCTTCATCACC-3', using an initial denaturation step at 94°C for 10 minutes, followed by 35 cycles of amplification in a three-step reaction that consisted of denaturation at 94°C for 30 seconds, annealing at 66°C for 30 seconds, and elongation at 72°C for 45 seconds, with a final extension step at 72°C for three minutes. The PCR product (10 μL) was digested by 4U of BsrI at 65°C overnight. The A allele is represented by a band of DNA with 445 bp and the G allele by two bands of DNA with 248 and 197 bp. The AG-heterozygous genotype has a combination of both alleles. All products were separated by electrophoresis on a 5% agarose gel containing EtBr and visualized under ultraviolet light. Each gel was read by two unbiased scientists unaware of the subject's disease status.

Statistical analysis was conducted using the PASW 17 (SPSS release 17.0; SPSS Inc., Chicago, IL). Continuous variables (age and BMI) were expressed as mean ± standard deviation (SD). Categorical variables (gender, genotype, and allele frequencies) were expressed as frequency (number) and the corresponding percentages (%). Testing for normality of the distribution of measurements was done using the Kolmogorov-Smirnov test or Shapiro-Wilk test. Student's unpaired t test was used to compare continuous values among cases and controls and any other subgroups. Categorical variables were compared using the chi-square (X2) test. The chi-square test was used to test the genotype distributions for deviation from Hardy-Weinberg equilibrium. Binary logistic regression analysis was used to estimate the relative risk of OA for each of the tested genotypes and haplotypes in the form of odds ratio (OR) and to identify independent prognostic factors for knee OA. A probability value of P < 0.05 was considered statistically significant. Based on a priori power analysis, it was estimated that at least 100 patients per group were needed to detect a 15% difference in allele frequencies to achieve a statistical power of 80% at a significance of 0.05.

## Results

The study included 100 patients with a mean age of 68.3 ± 6.0 years with primary knee OA and 100 controls with a mean age of 65.2 ± 8.1 years. The BMI of patients with OA (28.8 kg/m^2^) was higher, compared to controls (25.9 kg/m^2^) (P < 0.001). The demographics of the studied population are summarized in Table 1.

**Table 1:** Descriptive characteristics of the study sample SD: standard deviation, BMI: body mass index

	Controls	Cases	Overall	P-value
	Number (%)	Number (%)	Number (%)	
Gender				0.001
Female	53 (53.0)	76 (76.0)	129 (64.5)	
Male	47 (47.0)	24 (24.0)	71 (35.5)	
	Mean (SD)	Mean (SD)	Mean (SD)	P-value
Age (years)	65.2 (8.1)	68.3 (6.0)	66.8 (7.3)	0.006
BMI (kg/m^2^)	25.9 (1.7)	28.8 (2.3)	27.3 (2.5)	<0.001

Concerning radiological severity, 16% of OA patients were classified as K-L scale of 2, 72% on a scale of 3, and 12% on a scale of 4.

MMP-9 836A/G and MMP-13 -77A/G polymorphisms did not deviate from Hardy-Weinberg equilibrium (P > 0.05). In the distribution of the genotypes of the MMP-2 -1175G/A polymorphism, a marginal deviation from the Hardy-Weinberg equilibrium was observed (P = 0.05) for the total population, but not individually for patients and controls. The genotypic and allelic frequencies of MMP-2 -1175G/A, MMP-9 836A/G, and MMP-13 -77A/G polymorphisms are summarized in Table 2.

**Table 2:** Genotype and allelic frequencies OR: odds ratio, CI: confidence interval, SNP: single nucleotide polymorphism

	Controls	Cases	P-value	Crude OR (95%CI)
SNP	Number (%)	Number (%)		
MMP-2 -1175G/A				
GG	68 (68.0)	60 (60.0)		1
GA	26 (26.0)	32 (32.0)	0.295	1.39 (0.75-2.60)
AA	6 (6.0)	8 (8.0)	0.468	1.51 (0.50-4.60)
Dominant model				
GG	68 (68.0)	60 (60.0)		1
GA + AA	32 (32.0)	40 (40.0)	0.239	1.42 (0.79-2.53)
Recessive model				
GG + GA	94 (94.0)	92 (92.0)		1
AA	6 (6.0)	8 (8.0)	0.581	1.36 (0.45-4.08)
Allele frequencies				
G	0.81	0.76		1
A	0.19	0.24	0.224	1.35 (0.83-2.18)
MMP-9 836 A/G				
AA	48 (48.0)	40 (40.0)		1
AG	40 (40.0)	52 (52.0)	0.138	1.56 (0.87-2.81)
GG	12 (12.2)	8 (8.0)	0.658	0.80 (0.30-2.15)
Dominant model				
AA	48 (48.0)	40 (40.0)		1
AG + GG	52 (52.0)	60 (60.0)	0.256	1.38 (0.79-2.43)
Recessive model				
AA + AG	88 (88.0)	92 (92.0)		1
GG	12 (12.2)	8 (8.0)	0.349	0.64 (0.25-1.63)
Allele frequencies				
A	0.68	0.66		1
G	0.32	0.34	0.671	1.09 (0.72-1.66)
MMP-13 -77A/G				
AA	74 (74.0)	56 (56.0)		1
AG	26 (26.0)	40 (40.0)	0.021	2.03 (1.11-3.72)
GG	0 (0.0)	4 (4.0)	0.099	11.87 (0.62-224.97)
Dominant model				
AA	74 (74.0)	56 (56.0)		1
AG + GG	26 (26.0)	44 (44.0)	0.008	2.23 (1.23-4.06)
Recessive model				
AA + AG	100 (100.0)	96 (96.0)		1
GG	0 (0.0)	4 (4.0)	0.135	9.37 (0.50-176.44)
Allele frequencies				
A	0.87	0.76		1
G	0.13	0.24	0.005	2.11 (1.25-3.57)

For the MMP-2 -1175G/A and MMP-9 836A/G polymorphisms, there was no significant difference in genotype distribution between patients and controls (P = 0.495 and P = 0.213, respectively). Similarly, there was no significant difference in the frequencies of alleles (P = 0.223 and P = 0.670, respectively). However, for MMP-13 -77A/G polymorphism, the percentage of AA genotype was lower in OA patients (56%) in comparison to controls (74%) (P = 0.009), and the prevalence of the G allele was higher in OA cases (24%) compared to controls (13%) (P = 0.005).

In binary logistic analysis, OA was tested as the dependent variable, while age, gender, and BMI were tested as independent variables. The dominant model of the MMP-13 -77A/G (AG + GG versus AA) was associated with increased risk for knee OA (dominant model: OR = 2.290, 95%CI = 1.059-4.949, P = 0.035). The G allele in the MMP-13 rs2252070 locus was found to be a predictive factor for knee OA (OR = 2.351, 95%CI = 1.134-4.874, P = 0.022). Female gender and increased BMI were also predisposing factors for knee OA. Results from binary regression analysis are presented in Table 3.

**Table 3:** Results from binary regression analysis for the association between MMP-13 -77A/G SNP and risk of knee OA, adjusted for age, gender, and BMI MMP: matrix metalloproteinase, SNP: single nucleotide polymorphism, OA: osteoarthritis, BMI: body mass index, OR: odds ratio, CI: confidence interval

	Adjusted OR (95%CI)	P-value
MMP-13		
AG + GG versus AA	2.290 (1.059-4.949)	0.035
G allele versus A allele	2.351 (1.134-4.874)	0.022
Age	1.045 (0.992-1.100)	0.096
BMI	2.002 (1.627-2.462)	<0.001
Gender (men versus women)	4.244 (1.878-9.593)	0.001

## Discussion

To the best of our knowledge, this is the first study attempting to evaluate the role of the polymorphisms -1575G/A MMP-2 (rs243866), 836A/G MMP-9 (rs17576), and -77A/G MMP-13 (rs2252070) in the risk of knee OA in the Greek population. The results of this study suggest that the MMP-13 -77A/G SNP is a risk factor for knee OA in the Greek population. No association was detected between the MMP-2 -1575G/A and the MMP-9 836 A/G polymorphisms.

SNPs are the most common type of genetic variation found in humans. They are a natural part of genetic diversity and can occur throughout the genome. SNPs are usually present in at least 1% of the population. Genome-wide association studies (GWAS) often rely on the analysis of SNPs to identify links between specific genetic variants and diseases. Considering the heterogeneity and complexity of osteoarthritis, it is not surprising that these SNPs have different levels of association with OA. Some SNPs may be specific for OA (hip, knee, and hand) subtypes between different ethnic groups, but the results show a wide range of deviations [20].

MMP-2 is produced by various cell types within the joint, including chondrocytes and synovial cells, and it can contribute to inflammation by degrading ECM type IV collagen and releasing bioactive fragments that trigger synovial inflammatory responses. Elevated levels of MMP-2 have been observed in the synovial fluid and cartilage of OA patients, particularly in the advanced stages of the disease [21]. The present study concluded that the -1575G/A MMP-2 SNP does not affect the risk for knee OA, a finding that comes in agreement with the study by Barlas et al. [22], which found no association between MMP-2 -1306 C/T (rs243865) and knee OA risk in a Turkish population.

In articular cartilage, MMP-9 is produced by immune cells, such as monocytes and macrophages. MMP-9 is also secreted by articular chondrocytes at low levels, regulating MMP-9 expression by monocytes. Increased levels of MMP-9 have been found in the synovial fluid of OA patients [23]. In agreement with the results of the present study, the 836A/G MMP-9 SNP was not found to be related to knee OA in a Finnish population [24]. Moreover, another SNP in the promoter region of the MMP-9 gene (-1562C/T) was not associated with knee OA in Turkish populations [22].

MMP-13 is the most studied MMP in OA pathophysiology as it has a five- to 10-fold higher enzymatic activity than MMP-1 in the breakdown of type II collagen. Moreover, MMP-13 may degrade other types of ECM proteins playing an important role in normal ECM remodeling and reorganization in healthy cartilage [25]. MMP-13 overactivity leads to the progressive degradation of cartilage, resulting in the characteristic loss of cartilage thickness and quality seen in OA [26]. Elevated levels of MMP-13 have been observed in the synovial fluid, cartilage, and subchondral bone of OA patients, particularly in the advanced stages of the disease.

The -77A/G polymorphism is believed to influence the transcriptional activity of the MMP-13 gene. When the A allele is replaced by the G one, there is a decrease in the affinity for the transcription factor activator protein-1 (AP-1) responsible for tissue-specific MMP transcription [27]. Higher expression of MMP-13 can result in greater cartilage degradation, potentially contributing to OA progression. The present study has concluded that the AA genotype and the presence of the A allele are associated with a lower risk of knee OA in the Greek population (OR = 0.437, 95%CI = 0.202-0.944, P = 0.035). This finding contradicts the conclusions of another case-control study that suggested that the MMP-13 -77A/G polymorphism is associated with increased risk for knee OA and positively correlated with knee OA severity in the Chinese Han population (OR = 1.361, 95%CI = 1.151-1.569, P < 0.001). The A allele was associated with a 24% increased risk for knee OA [28].

The observed difference between the two studies may be attributed to several factors. The mean age and BMI of the studied population in the present study were significantly higher, suggesting that OA patients were older and more obese. The ratio of male gender was significantly lower in the present study. In our study, the majority of patients were classified as K-L score 3 (72%), while in the study of Sun et al. [28], most patients were classified as K-L score 2 (54.5%). All these facts raise the possibility that our study included patients with more severe knee OA. Moreover, the frequency of the A allele in the present study was 82%, while in the Chinese Han population, the frequency of the A allele was 36.9%, verifying the fact that the A allele is rarer in Asian populations [28]. In conclusion, the disagreement between the two studies can be explained by the racial and ethnic variations, different environmental interactions, and potential differences in knee OA severity in the patient group. It is important to note that the development and progression of OA are complex and involve interactions between genetic factors, environmental factors, and other genetic polymorphisms. The -77A/G polymorphism is just one piece of the puzzle, and its effects may be influenced by other genetic and environmental factors.

Two studies have been published in Greek populations, investigating the potential association of the MMP-13 -77A/G polymorphism with various diseases. Vairaktaris et al. [29] found that the presence of the A allele is not associated with oral cancer. A similar case-control study by Makrygiannis et al. [30] found no association between the MMP-13 -77A/G and large abdominal aortic aneurysms in the Greek population. In these two studies, the frequency of the A allele was 66.4% and 62.4%, respectively.

The present study has several limitations. First, the study population is relatively low, creating the possibility of type I or type II errors. The observed lack of association of -1575G/A MMP-2 and 836A/G MMP-9 polymorphisms may simply reflect that these SNPs have a minor influence in knee OA pathogenesis, which is too small to be detected with our study population, and a larger study population may be required. Second, we evaluated only one SNP of MMP-2, MMP-9, and MMP-13. It is possible that the other SNPs might also be associated with MMP expression levels. After all, the existence of external mechanical forces that may contribute to the development and progression of knee OA could not be ruled out.

## Conclusions

The present study indicated that the MMP-2 -1575G/A (rs243866) and MMP-9 836A/G (rs17576) polymorphisms are not associated with primary knee OA in the Greek population. The MMP-13 -77A/G had a significantly higher rate in the knee OA group compared to the control group in the Greek population. Additional research is needed to verify MMP-13 -77A/G as a significant risk factor for knee OA, evaluating this association in larger and different populations, in different joints, and to elucidate the role of this polymorphism in the pathogenesis of OA.
